# Variety and Harvesting Season Effects on Antioxidant Activity and Vitamins Content of *Citrus sinensis* Macfad

**DOI:** 10.3390/molecules20058287

**Published:** 2015-05-07

**Authors:** Vanessa Cardeñosa, João C. M. Barreira, Lillian Barros, Francisco J. Arenas-Arenas, José M. Moreno-Rojas, Isabel C. F. R. Ferreira

**Affiliations:** 1 Postharvest Technology and Agrifood Industry Area, Andalusian Institute of Agricultural and Fishering Research and Training (IFAPA) Alameda del Obispo, 14004 Córdoba, Spain; E-Mails: vanessa.cardenosa@juntadeandalucia.es (V.C.); josem.moreno.rojas@juntadeandalucia.es (J.M.M.-R.); 2 Mountain Research Center (CIMO), ESA, Polytechnic Institute of Bragança, Apartado 1172, 5301-855 Bragança, Portugal; E-Mails: jbarreira@ipb.pt (J.C.M.B.); lillian@ipb.pt (L.B.); 3 Centro IFAPA “Las Torres-Tomejil”, Instituto de Investigación y Formación Agraria y Pesquera, Ctra Sevilla—Cazalla Km. 12,2, 41200 Alcalá del Río (Sevilla), Spain; E-Mail: fjose.arenas@juntadeandalucia.es

**Keywords:** *Citrus sinensis*, variety, harvesting season, antioxidant activity, ascorbic acid, α-tocopherol

## Abstract

Five sweet orange (*Citrus sinensis* Osbeck) varieties cultivated in Huelva (Spain) and picked at two seasons during two consecutive years, were characterized for their antioxidant activity (free radicals scavenging activity, reducing power and lipid peroxidation inhibition) and vitamin content (vitamin E and vitamin C). The effects induced by sweet orange variety and stage of maturity were comprehensively compared by applying 2-way ANOVA and linear discriminant analysis. The results indicated higher differences in antioxidant activity and vitamin contents in response to the effect of the harvesting season, when compared to the effect of sweet orange variety. Nevertheless, the results observed in 2012 showed less marked differences among the assayed sweet orange varieties. Either way, it might be concluded that oranges sampled in January show the highest antioxidant activity and vitamin contents. Furthermore, concerning the properties evaluated in this work, all sweet orange varieties represent good alternatives, except for Rhode Summer, which would not be the preferable choice as a target to enhance sweet orange overall characteristics.

## 1. Introduction

In recent decades, researchers and consumers have become increasingly interested in a healthier diet, increasing the intake of fruit and vegetables. This is mainly justified because these foods are an important source of bioactive compounds. In fact, several authors had already suggested that a diet rich in antioxidants is directly associated with the prevention of human diseases, like several kinds of cancer, diabetes, cardiovascular diseases, neurological disorders, among others [1,2,3]. All these pathologies have the same underlying feature of being related with the overproduction of free radicals in the organism [1]. These chemical species are generally unstable and very reactive atoms or molecules with one or more unpaired electrons. They are usually divided in three classes: (1) reactive oxygen species; (2) reactive nitrogen species; and (3) reactive sulfur species. They are produced as a normal part of aerobic metabolism but external factors such as drugs, environmental pollutants, radiation, smoking, industrial solvents, pesticides and ozone can promote their production in the organism [4]. For normal organism functioning, it is essential to maintain the equilibrium between the production of these “reactive species” (RS) and their neutralization by antioxidant defenses, but, if the balance tends to the overproduction of RS, it is said that the organism is in oxidative stress.

Citrus fruits are an important source of antioxidant compounds to prevent oxidative stress. In particular, sweet orange fruits are considered to be rich sources of antioxidants, including vitamin C, phenolic compounds and carotenoids [5,6,7,8]. Among these compounds, the intake of ascorbic acid (vitamin C) is critical because the human organism cannot synthesize it. Moreover, ascorbic acid is associated with a reduced incidence of various oxidative stress-related diseases such as heart disease, stroke, cancer, several neurodegenerative diseases and cataractogenesis [9,10]. On the other hand, the intake of vitamin E (tocopherols), also found in fruits and vegetables, is believed to prevent against degenerative malfunctions too, mainly cancer and cardiovascular diseases due to its role as a scavenger of free radicals [11,12].

Regarding citrus fruits production in Europe, Spain is the largest producer and exporter of sweet orange fruit. However, the strong market competitiveness demands the development of new varieties in order to improve some aspects of the crop, such as productivity and fruit quality, with clear benefits for the sweet orange growers. Previously, several citrus varieties have been found to display significantly higher levels of antioxidant activity than others, and the individual content in flavonoids and phenolic acids have been found to differ among varieties as well [13,14]. Nevertheless, studies regarding the influence of harvesting season are rather scarce. Accordingly, the main purpose of the present work was to verify possible differences in the antioxidant quality of different sweet orange varieties (Barnfield, Chislet, Lane Late, Navel Powell and Rhode Summer) grown in western Andalusia, but also evaluating if the harvesting season (January or April) may affect that quality. In order to validate the results, samples were harvested in two consecutive years. Finding the most suitable variety, as well as the harvesting season stage, that optimizes the antioxidant potential of this fruit might represent a valuable feature for sweet orange growers. 

## 2. Results and Discussion

The EC_50_ values obtained for each antioxidant assay, as well as the levels of ascorbic acid and α-tocopherol are presented in Table 1. The assayed orange variety (OV) showed antioxidant activity in all the performed assays, with the lowest EC_50_ values being obtained for β-carotene bleaching inhibition.

**Figure 1:**
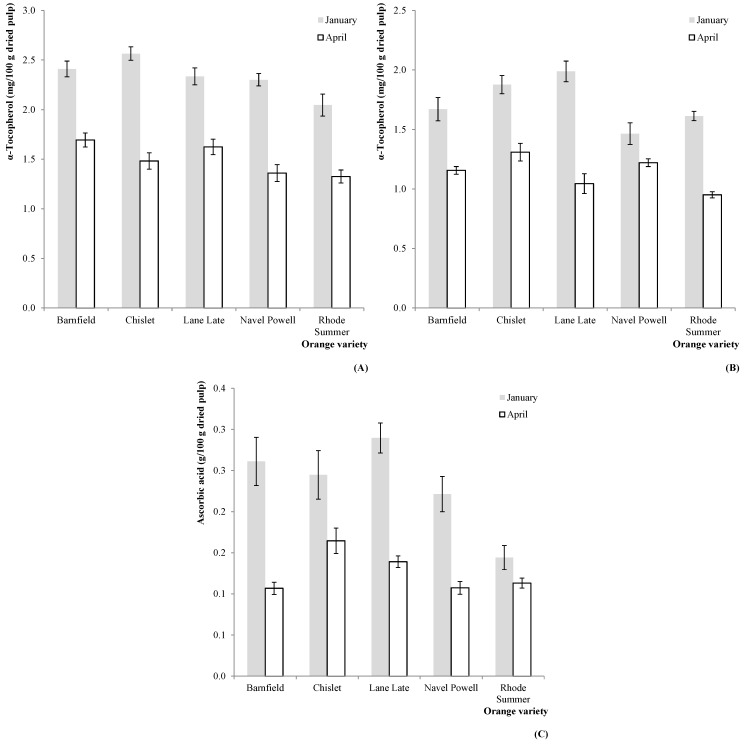
Estimated marginal mean plots representing the effect of harvesting season (HS) on the antioxidant vitamins of *C. sinensis* fruits. (**A**) α-tocopherol (2011); (**B**) α-tocopherol (2012); (**C**) ascorbic acid (2011).

**Table 1:** Antioxidant properties (EC_50_ values in mg/mL) and major antioxidant compounds divided by sweet orange variety (OV) and harvesting season (HS). The results are presented as mean ± SD.

		DPPH Scavenging Activity	Reducing Power	Lipid Peroxidation Inhibition		α-Tocopherol (mg/100 g of dry pulp)	Ascorbic Acid (g/100 g of dry pulp)
				TBARS Formation Inhibition	β-Carotene Bleaching Inhibition		
2011							
Orange variety (OV)	Barnfield	9 ± 2	2.5 ± 0.4	2.0 ± 0.5	1.3 ± 0.3	2.1 ± 0.4	0.18 ± 0.05
	Chislet	10 ± 2	3.0 ± 0.2	2.1 ± 0.2	0.4 ± 0.1	2.0 ± 0.5	0.20 ± 0.05
	Lane Late	9 ± 2	2.5 ± 0.3	2.1 ± 0.2	0.4 ± 0.1	2.0 ± 0.4	0.21 ± 0.05
	Navel Powell	9 ± 2	2.6 ± 0.5	2.5 ± 0.5	0.4 ± 0.1	1.8 ± 0.5	0.16 ± 0.05
	Rhode Summer	11 ± 1	3.0 ± 0.1	4.0 ± 0.2	0.5 ± 0.1	1.7 ± 0.4	0.13 ± 0.02
*p*-value (n = 18)	Tukey’s test	<0.001	<0.001	<0.001	<0.001	0.081	0.001

Harvesting season (HS)	January	8 ± 1	2.4 ± 0.3	3 ± 1	0.6 ± 0.2	2.3 ± 0.2	0.23 ± 0.05
	April	11 ± 1	3.0 ± 0.1	2 ± 1	0.6 ± 0.3	1.5 ± 0.2	0.13 ± 0.02
*p*-value (n = 45)	*t-*student’s test	<0.001	<0.001	0.088	0.696	<0.001	<0.001
*p*-value (n = 90)	OV × HS	<0.001	<0.001	<0.001	<0.001	<0.001	<0.001
2012							
Orange variety (OV)	Barnfield	7.8 ± 0.1	2.6 ± 0.1	4.3 ± 0.2	0.7 ± 0.2	1.4 ± 0.3	0.22 ± 0.03
	Chislet	7.4 ± 0.5	2.5 ± 0.2	3.8 ± 0.4	0.4 ± 0.2	1.6 ± 0.3	0.21 ± 0.02
	Lane Late	7.1 ± 0.5	2.4 ± 0.2	4 ± 1	0.7 ± 0.1	1.5 ± 0.5	0.25 ± 0.03
	Navel Powell	8.0 ± 0.1	2.6 ± 0.1	4 ± 1	0.7 ± 0.3	1.3 ± 0.1	0.20 ± 0.02
	Rhode Summer	7.0 ± 0.3	2.4 ± 0.2	4 ± 1	0.4 ± 0.1	1.3 ± 0.3	0.16 ± 0.02
*p*-value (n = 18)	Tukey’s test	<0.001	<0.001	0.600	<0.001	0.040	<0.001

Harvesting season (HS)	January	7.1 ± 0.5	2.4 ± 0.1	3.2 ± 0.5	0.5 ± 0.2	1.7 ± 0.2	0.21 ± 0.04
	April	7.8 ± 0.3	2.6 ± 0.1	5.2 ± 0.5	0.6 ± 0.3	1.1 ± 0.1	0.20 ± 0.03
*p*-value (n = 45)	*t-*student’s test	<0.001	<0.001	<0.001	0.022	<0.001	0.058
*p*-value (n = 90)	OV × HS	<0.001	<0.001	<0.001	<0.001	<0.001	0.038

The interaction effect between OV and HS was evaluated to understand if differences in antioxidant profiles or vitamin levels are specific of a determined OV or HS. The reported results are presented as the mean value of each OV for both HS, as well as the mean value of each HS, comprising values for all OV. Every time the interaction among factors (OV × HS) was significant (*p* < 0.05), acting itself as a source of variability, multiple comparison tests could not be performed. In these cases, the presented conclusions were drawn from the estimated marginal mean (EMM) plots obtained in each case. The results obtained for HS were compared using a simple *t*-test for equality of means (after checking the equality of variances through a Levene’s test), since there were fewer than three groups.

As it might be depicted from Table 1, the interaction OV × HS was significant in all cases, not allowing any multiple comparison tests. Nevertheless, some particular tendencies became evident from the analysis of the correspondent EMM plots: the α-tocopherol (in 2011, Figure 1A and 2012, Figure 1B) and ascorbic acid (in 2011, Figure 1C) levels were higher in samples collected in January for all the tested OV.

**Figure 2:**
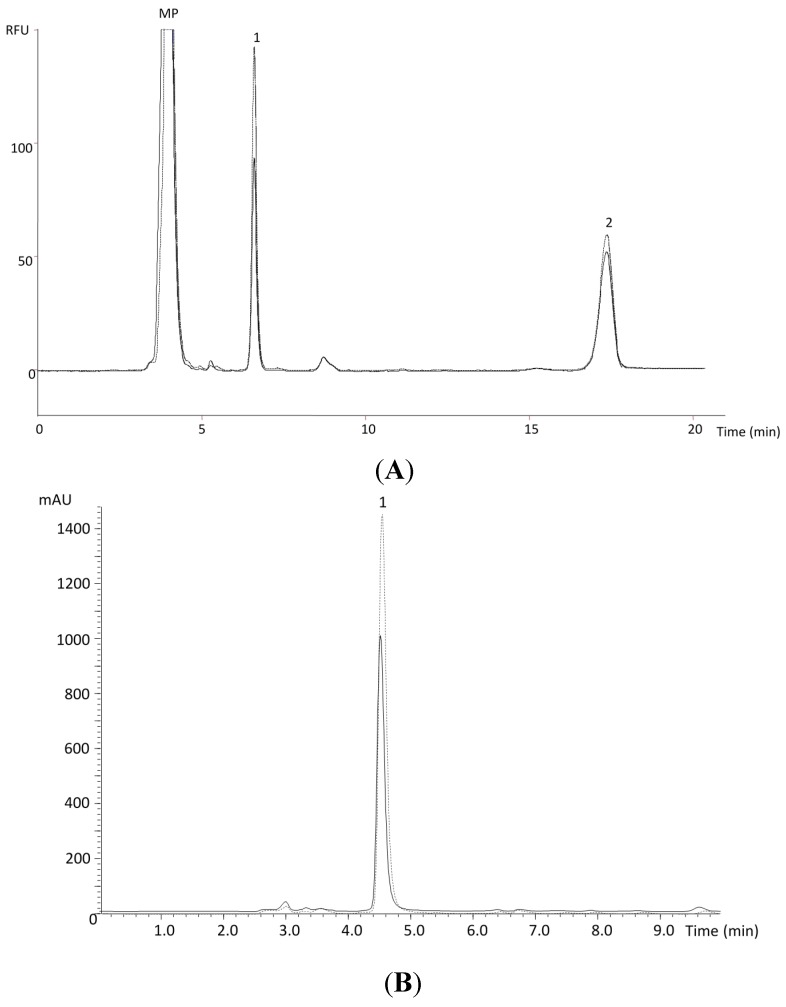
HPLC chromatograms of α-tocopherol [(**A**), MP—mobile phase; 1—α-tocopherol; 2—tocol] and ascorbic acid [(**B**), 1—ascorbic acid] in the dried pulp of Chislet variety for the year 2011 (----- January and ^______^ April).

Exemplifying chromatograms (Chislet variety) representing the results obtained for α-tocopherol (Figure 2A) and ascorbic acid (Figure 2B) are shown to highlight the upper amounts obtained in January 2011.

These higher levels in antioxidant compounds were reflected by the measured antioxidant activity, as it can be deduced from the results obtained for DPPH scavenging activity (in 2011, Figure 3A), reducing power (in 2011, Figure 3B and 2012, Figure 3C) and TBARS formation inhibition (in 2012, Figure 3D).

**Figure 3:**
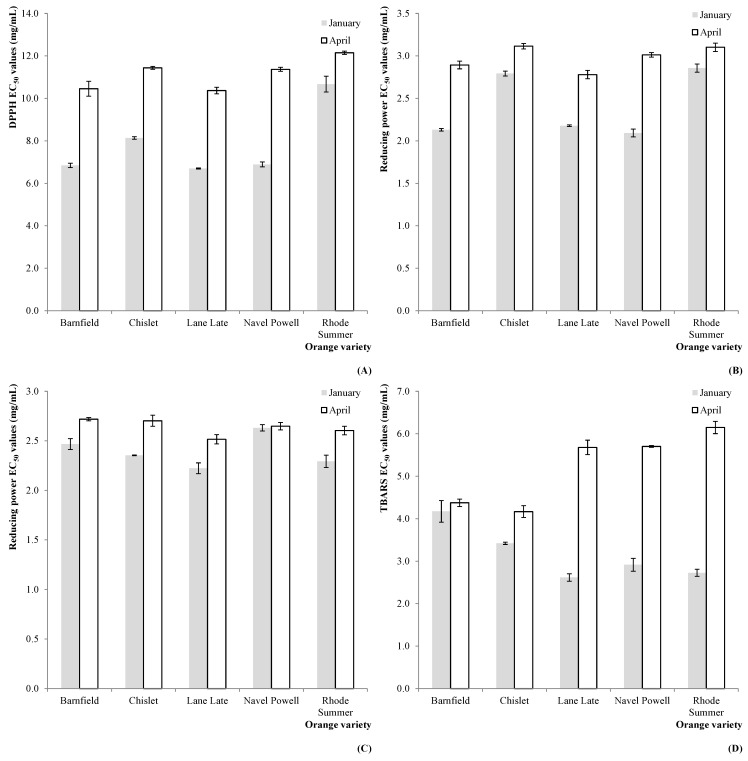
Estimated marginal mean plots representing the effect of HS on the antioxidant properties of *C. sinensis* fruits: (**A**) DPPH scavenging activity (2011); (**B**) reducing power (2011); (**C**) reducing power (2012); and (**D**) TBARS formation inhibition (2012).

In what concerns the OV effect, some tendencies could also be identified; namely, the lower levels of ascorbic acid in Rhode Summer (in 2012, Figure 4A), and the weaker antioxidant activity verified for this variety for DPPH scavenging activity (in 2011, Figure 4B) and TBARS formation inhibition (in 2011, Figure 4C). Other identified tendency was the lower β-carotene bleaching inhibition shown by the Barnfield variety (in 2011, Figure 4D).

**Figure 4:**
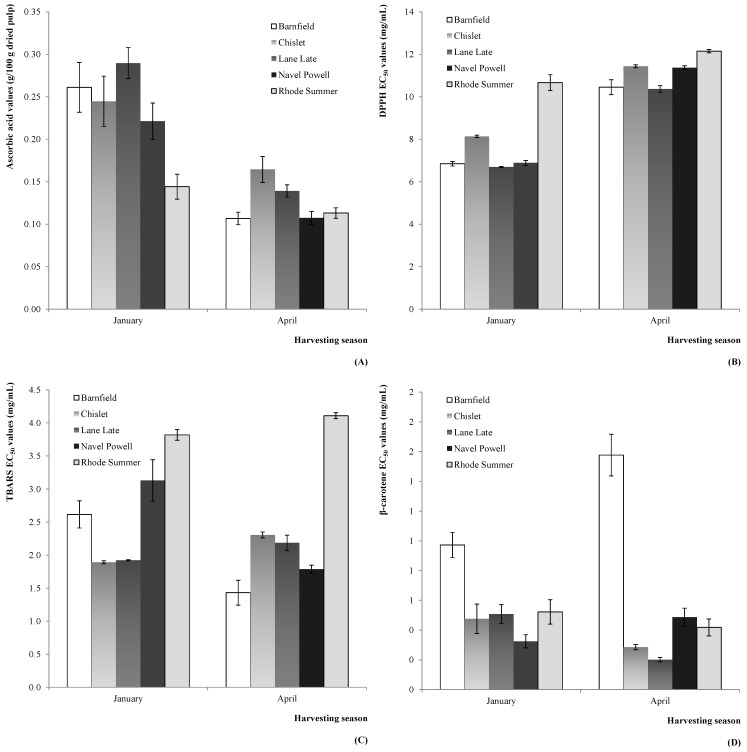
Estimated marginal mean plots representing the effect of OV on the antioxidant parameters of *C. sinensis* fruits: (**A**) ascorbic acid (2012); (**B**) DPPH scavenging activity (2011); (**C**) TBARS formation inhibition (2011); and (**D**) β-carotene bleaching inhibition (2011).

In general, the antioxidant activity, together with the levels of vitamins showed a higher variation in response to the effect of the HS when compared to the effect of OV. In fact, the harvesting season has been previously reported as inducing significant changes in sweet orange [15], as in other *Citrus* species [16] antioxidant properties.

**Figure 5:**
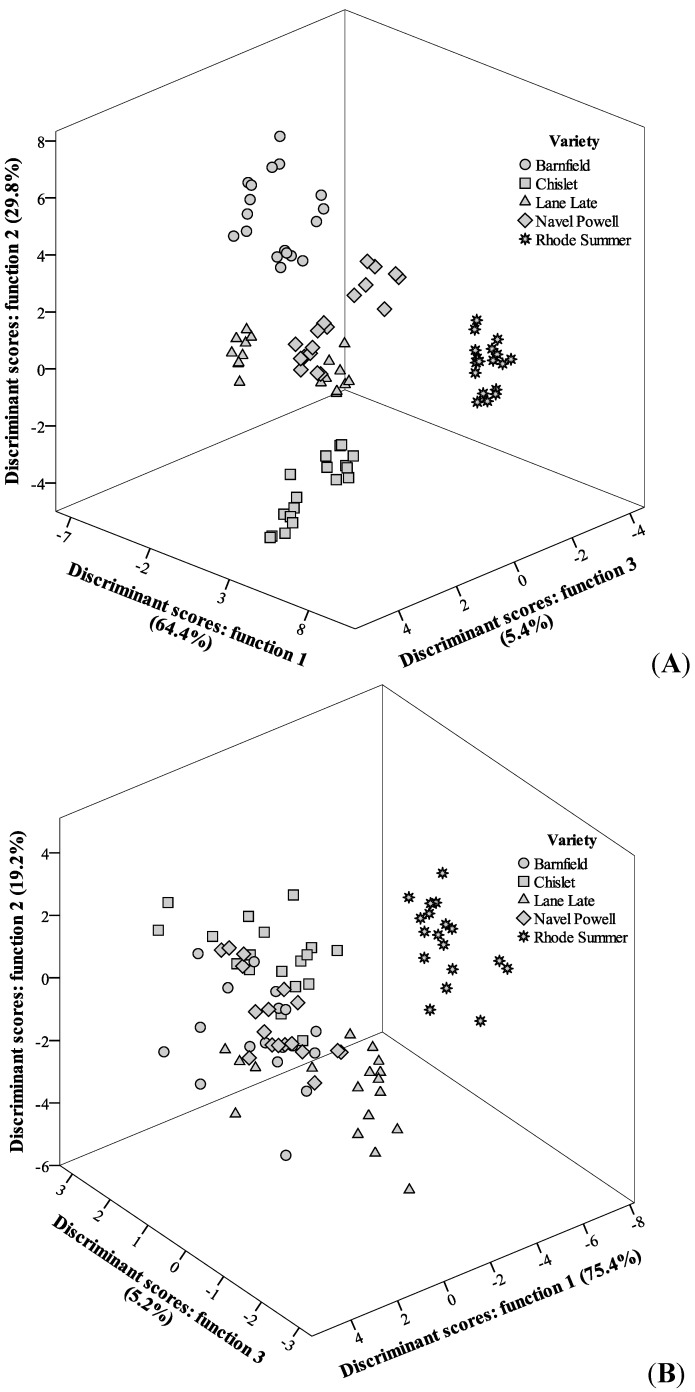
Discriminant scores scatter plot of the canonical functions defined for antioxidant parameters results according with OV for the years 2011 (**A**) and 2012 (**B**).

Aiming to understand the concerted effects of OV and HS on the antioxidant activity and vitamin amounts, two linear discriminant analyses (LDA) were applied, one for each single year. The significant independent variables (results for antioxidant activity assays and antioxidant compound levels) were selected using the stepwise method of the LDA, by evaluating the Wilks’ λ test results. Only those variables with a statistically significant classification performance (*p* < 0.05) were kept in the analysis.

In the case of OV effect, and starting with the analysis for year 2011, four significant functions were defined, from which the first three were plotted (Figure 5), including 99.6% of the results variance (function 1: 64.4%; function 2: 29.8%; function 3: 5.4%).

As it can be immediately depicted from the discriminant scores distribution, the tested groups (Barnfield, Chislet, Lane Late, Navel Powell and Rhode Summer) were almost completely separated. Function 1 was mostly correlated with DPPH scavenging activity and TBARS formation inhibition, contributing to the separation of Rhode Summer variety, which presented higher EC_50_ values for those two assays (Table 1). Function 2, more strongly correlated with β-carotene bleaching inhibition, had a higher effect on the separation of Barnfield variety, which had higher EC_50_ values in that assay. Function 3 was more correlated with β-carotene bleaching inhibition and TBARS formation inhibition but it did not contribute in great extent to separate any of the varieties. In terms of classification performance, the corresponding contingency matrix (Table 2) gave values of sensitivity and overall specificity of 89% within the leave-one-out cross-validation procedure, which may be considered as acceptable values. It is interesting to verify that only Lane Late and Navel Powell showed misclassified cases (6 Lane Late were classified as Navel Powell and 4 Navel Powell were classified as Lane Late).

**Table 2:** Contingency matrix obtained using linear discriminant analysys based on antioxidant activity and antioxidant contents of sweet orange varieties (OV).

OV	Predicted Group Membership					Total	Sensitivity (%)
	Barnfield	Chislet	Lane Late	Navel Powell	Rhode Summer		
2011							
Barnfield	18	0	0	0	0	18	100
Chislet	0	18	0	0	0	18	100
Lane Late	0	0	12	6	0	18	67
Navel Powell	0	0	4	14	0	18	78
Rhode Summer	0	0	0	0	18	18	100
total	18	18	16	20	18	90	89
Specificity (%)	100	100	75	70	100	89	
2012							
Barnfield	11	1	0	6	0	18	61
Chislet	1	15	2	0	0	18	83
Lane Late	2	0	16	0	0	18	89
Navel Powell	6	0	0	12	0	18	67
Rhode Summer	0	0	0	0	18	18	100
total	20	16	18	18	18	90	80
Specificity (%)	55	94	89	67	100	81	

Concerning the results for samples harvested during 2012, the discriminant model also selected four significant functions, which included 100.0% of the observed variance. The graph representation (Figure 5) of the three first functions (function 1: 75.4%, function 2: 19.2%, function 3: 5.2%) did not show the assayed groups as separated as in the case of year 2011, but some differences among OV are still evident. Function 1 was mostly correlated with ascorbic acid content, contributing to the separation of Rhode Summer variety (which presented lower levels of this vitamin). Function 2 was also more highly correlated with ascorbic acid, favoring the separation of Lane Late variety (which presented the maximum levels of this vitamin). Function 3 correlated better with β-carotene bleaching inhibition, contributing to separate Chislet variety (which presented low EC_50_ values for this antioxidant activity assay). The classification ability was not as high as that obtained for the year 2011 (Table 2), but the values for sensitivity (80%) and overall specificity (81%) within the leave-one-out cross-validation procedure, may still be considered as acceptable values. Despite some misclassified samples, it is relevant to verify that, as it happened for the results from the year 2011, all Rhode Summer samples were correctly classified, which might indicate that this variety has, in fact, differences in its antioxidant activity when compared to the other assayed varieties.

Regarding the combined effect of HS, samples harvested in each one of the months were clearly separated and classified with 100% accuracy, with the same result being obtained for both years. A single function was defined in each case (the number of defined functions equals the number of levels for a determined factor less one), more correlated with α-tocopherol content in year 2011, and α-tocopherol content and TBARS formation inhibition in year 2012.

## 3. Experimental Section

### 3.1. Samples

Five sweet orange varieties (Barnfield, Chislett, Lane Late, Navel Powell and Rhode Summer), all belonging to Navel group (midseason varieties) were grown in western Andalusia (Huelva, Spain) on a plot of 9000 m^2^ (37°39′56′′N, 6°35′10.06′′W; UTM coordinates: X: 712,306.23 Y: 417,095.95).

The oranges were harvested in two harvesting seasons, at the beginning and end of the season (January and April). Seven-year-old trees grafted on Carrizo citrange were selected. The experimental design was a randomized block with four replications and an experimental unit of three trees per variety. The references to the test plot climatological data were collected from the nearest station weather. The weather showed for this period was Mediterranean weather type with a regime of low seasonal rainfall and a marked summer drought. The annual rainfall was 688 mm and evapotranspiration (ET_0_) 1400 mm.

The production yield of each tree presented similar values (≈73 kg/tree) for all varieties. Samples harvesting was performed in two consecutive years (2011 and 2012). After being collected and subsequently transported from the field to the lab (keeping cold), the samples were stored at 8 ºC until further use. For each variety and harvesting season, 10 oranges were pooled and the mixed pulp was divided in three similar amounts. Fruit’s pulp was lyophilized (FreeZone 4.5 model 7750031, Labconco, Kansas City, MO, USA), reduced to a fine dried powder (20 mesh), mixed to obtain homogenous samples, and stored in a desiccator, protected from light, until further analysis.

All samples reached the minimum requirements for quality standards imposed by regulation (color index > 6; maturity index > 6.5; equatorial diameter > 53mm and over 33% juice) (EC Regulation No. 1221/2008). 

### 3.2. Standards and Reagents

Ethyl acetate 99.8% and n-Hexane 95% were of HPLC grade from Fisher Scientific (Lisbon, Portugal). Trolox (6-hydroxy-2,5,7,8-tetramethylchroman-2-carboxylic acid), α-tocopherol and ascorbic acid standards were purchased from Sigma (St. Louis, MO, USA). Racemic tocol, 50 mg/mL, was purchased from Matreya (Pleasant Gap, PA, USA). 2,2-Diphenyl-1-picrylhydrazyl (DPPH) was obtained from Alfa Aesar (Ward Hill, MA, USA). Methanol and all other chemicals were of analytical grade and purchased from common sources. Water was treated in a Milli-Q water purification system (TGI Pure Water Systems, Greenville, SC, USA).

### 3.3. Antioxidant Activity Evaluation

Each lyophilized sample (0.5 g) was extracted by stirring with 20 mL of methanol/water (80:20, *v/v*) for 1 h and subsequently filtered through Whatman No. 4 paper. The residue was then extracted with 20 mL of methanol/water (80:20, *v/v*) for 1 h. The combined hydro-alcoholic extracts were evaporated at 40 °C (rotary evaporator Büchi R-210, Flawil, Switzerland) to dryness and re-dissolved in methanol/water (80:20, *v/v*) for antioxidant activity assays (40 mg/mL). Successive dilutions were made from the stock solution and submitted to the *in vitro* assays already described by Martins *et al.* [17], to evaluate the antioxidant activity of the samples. The sample concentrations (mg/mL) providing 50% of antioxidant activity or 0.5 of absorbance (EC_50_) were calculated from the graphs of antioxidant activity percentages (DPPH, β-carotene/linoleate and TBARS assays) or absorbance at 690 nm (ferricyanide/Prussian blue assay) against sample concentrations. Trolox was used as a positive control and the assays were performed at room temperature with dimmed light.

#### 3.3.1. Reducing Power by Ferricyanide/Prussian Blue Assay

The extract solutions with different concentrations (0.5 mL) were mixed with sodium phosphate buffer (200 mmol/L, pH 6.6, 0.5 mL) and potassium ferricyanide (1% *w/v*, 0.5 mL). The mixture was incubated at 50 °C for 20 min, and trichloroacetic acid (10% *w/v*, 0.5 mL) was added. The mixture (0.8 mL) was poured in the 48 wells plate, the same with deionized water (0.8 mL) and ferric chloride (0.1% *w/v*, 0.16 mL), and the absorbance was measured at 690 nm in ELX800 Microplate Reader (Bio-Tek Instruments, Inc.; Winooski, VT, USA).

#### 3.3.2. DPPH Radical-Scavenging Activity Assay

This methodology was performed using the Microplate Reader mentioned above. The reaction mixture on 96-well plate consisted of a solution by the well of the extract solutions with different concentrations (30 μL) and methanolic solution (270 μL) containing DPPH radicals (6 × 10^−5^ mol/L). The mixture was left to stand for 30 min in the dark, and the absorption was measured at 515 nm. The radical scavenging activity (RSA) was calculated as a percentage of DPPH discoloration using the equation: %RSA = [(A_DPPH_ − A_S_)/A_DPPH_] × 100, where A_S_ is the absorbance of the solution containing the sample, and A_DPPH_ is the absorbance of the DPPH solution.

#### 3.3.3. Inhibition of β-Carotene Bleaching or β-Carotene/Linoleate Assay

A solution of β-carotene was prepared by dissolving β-carotene (2 mg) in chloroform (10 mL). Two milliliters of this solution were pipetted into a round-bottom flask. The chloroform was removed at 40 °C under vacuum and linoleic acid (40 mg), Tween 80 emulsifier (400 mg), and distilled water (100 mL) were added to the flask with vigorous shaking. Aliquots (4.8 mL) of this emulsion were transferred into test tubes containing extract solutions with different concentrations (0.2 mL). The tubes were shaken and incubated at 50 °C in a water bath. As soon as the emulsion was added to each tube, the zero time absorbance was measured at 470 nm. β-Carotene bleaching inhibition was calculated using the following equation: (Absorbance after 2 h of assay/initial absorbance) × 100.

#### 3.3.4. Thiobarbituric Acid Reactive Substances (TBARS) Assay

Porcine (*Sus scrofa*) brains were obtained from officially slaughtered animals, dissected, and homogenized with a vortex in an ice-cold Tris-HCl buffer (20 mM, pH 7.4) to produce a 1:2 w/v brain tissue homogenate, which was centrifuged at 3000 *g* for 10 min. An aliquot (100 μL) of the supernatant was incubated with the different concentrations of the sample solutions (200 μL) in the presence of FeSO_4_ (10 mM; 100 μL) and ascorbic acid (0.1 mM; 100 μL) at 37 °C for 1 h. The reaction was stopped by the addition of trichloroacetic acid (28% w/v, 500 μL), followed by thiobarbituric acid (TBA, 2%, w/v, 380 μL), and the mixture was then heated at 80 °C for 20 min. After centrifugation at 3000 *g* for 10 min to remove the precipitated protein, the color intensity of the malondialdehyde (MDA)-TBA complex in the supernatant was measured by its absorbance at 532 nm. The inhibition ratio (%) was calculated using the following formula: Inhibition ratio (%) = [(A − B)/A] × 100%, where A and B were the absorbance of the control and the sample solution, respectively.

### 3.4. Vitamins Content

#### 3.4.1. Tocopherols

BHT solution in hexane (10 mg/mL; 100 μL) and IS solution in hexane (tocol; 50 μg/mL; 400 μL) were added to the sample prior to the extraction procedure. In order to avoid tocopherols oxidation, the samples were protected from light and heat. The samples (~500 mg) were homogenized with methanol (4 mL) by vortex mixing (1 min). Subsequently, hexane (4 mL) was added and again vortex mixed for 1 min. After that, saturated NaCl aqueous solution (2 mL) was added, the mixture was homogenized (1 min), centrifuged (5 min, 4000 *g*) and the clear upper layer was carefully transferred to a vial. The sample was re-extracted twice with hexane. The combined extracts were taken to dryness under a nitrogen stream, redissolved in 2 mL of *n*-hexane, dehydrated with anhydrous sodium sulfate, filtered through 0.2 µm nylon filters from Whatman, transferred into a dark injection vial prior to the analysis [18]. Analysis was performed in a HPLC system consisted of an integrated system with a pump (Knauer, Smartline system 1000, Berlin, Germany), degasser system (Smartline manager 5000), an auto-sampler (AS-2057 Jasco, Easton, MD, USA), and a fluorescence detector (FP-2020; Jasco) programmed for excitation at 290 nm and emission at 330 nm. The compounds were identified by chromatographic comparisons with authentic standards. Quantification was based on the fluorescence signal response of each standard, using the IS (tocol) method and by using calibration curves obtained from commercial standards of each compound. The results were expressed in mg per 100 g of dry pulp.

#### 3.4.2. Ascorbic Acid

Samples (~0.5 g) were extracted by stirring with 10 mL of meta-phosphoric acid (25 °C at 150 rpm) protected from light for 45 min and subsequently filtered through Whatman No. 4 paper [19]. Ascorbic acid was analyzed by ultra-fast liquid chromatography (UFLC, Shimadzu 20A series, Shimadzu Corporation, Kyoto, Japan) coupled with a photodiode array detector (PDA) as previously optimized and described by the authors [19]. The quantification was made by comparison of the area of the peaks recorded at 245 nm with the calibration curve obtained from commercial standard of L-ascorbic acid. The results were expressed in g per 100 g of dry pulp.

### 3.5. Statistical Analysis

For each sample, all the extractions were performed in triplicate; each replicate was also measured three times. Data were expressed as mean ± standard deviation. All statistical tests were performed at a 5% significance level using IBM SPSS Statistics for Windows, version 22.0. (IBM Corp., Armonk, NY, USA).

An analysis of variance (ANOVA) with type III sums of squares was performed using the GLM (General Linear Model) procedure of the SPSS software. The dependent variables were analyzed using 2-way ANOVA, with the factors “orange variety” (OV) and “harvesting season” (HS). When a statistically significant interaction (OV × HS) was detected, the two factors were evaluated simultaneously by the estimated marginal means plots for all levels of each single factor. Alternatively, if no statistical significant interaction was verified, means were compared using Tukey’s honestly significant difference (HSD) multiple comparison test.

Stepwise Linear Discriminant Analysis (LDA) was used to verify if differences found for antioxidant activity and bioactive compound contents were high enough to discriminate the evaluated OV (Barnfield, Chislet, Lane Late, Navel Powell and Rhode Summer) as also to conclude which of the HS (January or April) optimizes the indicated properties. A stepwise technique, using the Wilks’s λ method with the usual probabilities of *F* (3.84 to enter and 2.71 to remove) was applied to select variables. This procedure followed a combination of forward selection and backward elimination steps; *i.e.*, before a new variable is selected to be included, it is verified whether all previously selected variables remain significant. The combination of variables is defined in a way that the first function furnishes the most general discrimination between groups, the second provides the second most, and so on. To verify which canonical discriminant functions were significant, the Wilks’ λ test was applied. To keep a more realistic data modulation, a leave-one-out cross-validation procedure was carried out to assess the model performance. 

## 4. Conclusions

Overall, the results obtained in this work proved that the differences in antioxidant activity and related compounds naturally existing in sweet orange varieties are overcome by the effects of the harvesting season. In fact, some particular compounds, especially α-tocopherol, as well as specific antioxidant activity indicators, such as the TBARS formation inhibition, are strongly altered by the harvesting season of sweet orange. When comparing the results of consecutive years, changes observed in 2012 showed less marked differences among the studied sweet orange varieties. Nevertheless, Rhode Summer variety was the less active variety in both years (as confirmed in the LDA), allowing saying that this would not be the preferable choice as a target to enhance the overall characteristics of oranges. Concerning the harvesting season, January resulted in a better option than April, when considering either antioxidant activity or vitamin contents.
